# Injury-Driven Structural and Molecular Modifications in Nociceptors

**DOI:** 10.3390/biology14070788

**Published:** 2025-06-29

**Authors:** Mario García-Domínguez

**Affiliations:** 1 Program of Immunology and Immunotherapy, CIMA-Universidad de Navarra, 31008 Pamplona, Spain; mgdom@unav.es; 2 Department of Immunology and Immunotherapy, Clínica Universidad de Navarra, 31008 Pamplona, Spain; 3 Centro de Investigación Biomédica en Red de Cáncer (CIBERONC), 28029 Madrid, Spain

**Keywords:** nerve injury, nociceptor, pain, plasticity, excitability, nerve remodelling

## Abstract

Peripheral tissue injury initiates a multifaceted cascade of structural and molecular modifications within nociceptors, the primary sensory neurons tasked with detecting noxious stimuli. These alterations play a crucial role in the induction and maintenance of pain states, encompassing acute and chronic pain. Structural remodeling includes alterations in axonal architecture, dendritic morphology, and synaptic connectivity, collectively impacting nociceptor excitability and functional integration. Simultaneously, molecular adaptations comprise the regulation of ion channels, receptor expression, and intracellular signaling pathways, as well as transcriptional reprogramming that modulates nociceptive signaling. This review synthesizes current evidence regarding the cellular and molecular bases of injury-induced plasticity in nociceptors, identifying prospective targets for therapeutic intervention to counteract maladaptive sensitization. Elucidating these processes is critical for the advancement of pain treatment strategies and for enhancing clinical outcomes in individuals experiencing neuropathic pain secondary to tissue injury.

## Simple Summary

Following tissue injury, nociceptors (the primary sensory neurons responsible for detecting pain) undergo extensive structural and functional modifications. These changes are pivotal in the development and maintenance of both acute and chronic pain. Structurally, injury leads to alterations in the morphology and synaptic connectivity of nociceptors, which influence their excitability and patterns of neural communication. Simultaneously, molecular adaptations occur, such as changes in the expression and function of ion channels, receptors, and intracellular signaling pathways, as well as shifts in gene transcription that modulate nociceptive signal processing. This review consolidates current insights into the mechanisms underlying nociceptor plasticity after injury.

## 1. Introduction

The International Association for the Study of Pain (IASP) describes pain as “an unpleasant sensory and emotional experience often associated with actual or potential tissue damage” [1,2]. This definition underscores the complex nature of pain, encompassing both physical and emotional components, and usually reflecting underlying health conditions that require clinical intervention [3,4,5]. Pain perception is initiated by the activation of nociceptors, specialized sensory neurons localized within peripheral tissues such as the skin, musculature, and internal organs [6]. Nociceptors, specialized in detecting noxious stimuli such as mechanical pressure, extreme thermal conditions, and chemical alterations indicative of potential tissue injury [7], express many transduction molecules and ion channels that mediate the conversion of these stimuli into electrical signals [8]. These signals are transmitted centrally to the spinal cord and brain, where they are processed and interpreted as the subjective experience of pain. Importantly, nociceptors exhibit considerable heterogeneity in both structure and function, comprising distinct subpopulations characterized by variations in activation thresholds, neurochemical profiles, conduction velocities, and patterns of target tissue innervation [9]. This diversity underlies the complexity and specificity of nociceptive signaling.

Recent evidence challenges the traditional view of nociceptors as passive transmitters of noxious stimuli, revealing their plasticity and active involvement in the modulation of pain signaling under both physiological and pathological conditions [10]. Nociceptors are dynamic, responsive cells capable of adapting to changing environmental stimuli, especially in the context of injury, inflammation, or persistent pathological conditions [11]. In response to peripheral tissue injury, nociceptors undergo several molecular and structural changes that collectively rewire their sensory capacity. At the transcriptional level, there is a strong expression of genes encoding voltage-gated sodium channels (VGSCs), notably Nav1.7, Nav1.8, and Nav1.9, which contribute to increased neuronal excitability by reducing the threshold required for the action potential initiation [12,13,14]. Concurrently, voltage-gated calcium channels (VGCCs), such as Cav2.2, become more abundant or more readily trafficked to the membrane, facilitating enhanced neurotransmitter release at central terminals [15]. Transient receptor potential (TRP) channels, such as TRPV1 and TRPA1, are sensitized through post-translational modifications, including phosphorylation by protein kinases (such as PKC, PKA, and MAPKs), which are triggered downstream of several pro-inflammatory mediators including prostaglandins, bradykinin, and nerve growth factor (NGF) [16]. This leads to increased neuronal excitability and reduced activation thresholds, enabling innocuous stimuli to induce pain (commonly termed allodynia) and potentiating the response to noxious stimuli, a phenomenon referred to as hyperalgesia [17].

Moreover, nociceptors mobilize receptors for pro-inflammatory cytokines and chemokines, including TNF-α, IL-1β, and CCL2, which further potentiate intracellular signaling cascades through activation of NF-κB and other transcription factors [18,19,20]. These pathways not only sustain peripheral sensitization but also influence some epigenetic regulators, promoting long-lasting changes in gene expression [21,22]. Axonal transport and translation of mRNAs encoding ion channels and signaling molecules within nociceptive terminals constitute a key mechanism for activity-dependent modulation of nociceptive signaling [23,24]. Furthermore, nociceptors increase the synthesis and release of neuropeptides such as substance P and calcitonin gene-related peptide (CGRP), which act both peripherally to promote vasodilation and immune cell recruitment, and centrally to enhance excitatory neurotransmission in the spinal dorsal horn [25,26].

Novel methodological innovations (like single-cell transcriptomics, in vivo calcium imaging, chemogenetics, and optogenetics) have deepened our ability to dissect nociceptor subtypes and their activity patterns with unprecedented resolution [27,28,29,30]. All of these technologies have identified injury-induced gene expression profiles that extend beyond typical stress responses, involving the activation of developmental and regenerative transcriptional pathways [31].

Collectively, these findings underscore the pivotal role of nociceptor molecular plasticity in driving the transition from acute nociceptive responses to persistent chronic pain states. The dynamic regulation of ion channel expression, receptor sensitization, intracellular signaling pathways, and neuropeptide release not only facilitates neuronal excitability but also contributes to long-term alterations in pain processing circuits. Understanding these adaptations provides valuable insight into the pathophysiology of chronic pain and identifies numerous potential molecular targets that could be exploited for the development of novel analgesic therapies aimed at preventing or reversing maladaptive nociceptive sensitization.

## 2. Structural and Functional Aspects of Nociceptors

**Figure 1:**
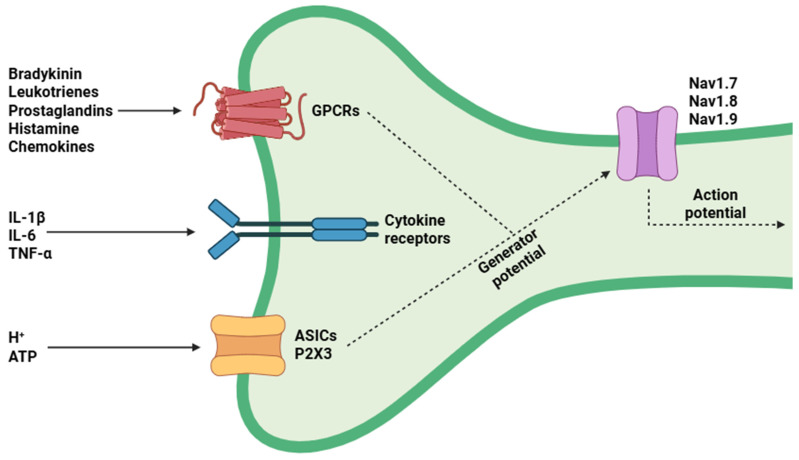
Nociceptors are specialized sensory neurons that can become activated under pathological conditions in response to a variety of stimuli, including thermal, mechanical, and chemical signals. Under physiological states, nociceptors detect noxious stimuli to initiate protective responses; however, during pathological states such as inflammation or nerve injury, their sensitivity can become markedly enhanced. Abbreviations: IL-1β (Interleukin 1 beta), IL-6 (interleukin-6), TNF-α (tumor necrosis factor alpha), ATP (adenosine triphosphate), GPCRs (G protein-coupled receptors), ASICs (acid-sensing ion channels), P2X3 (purinergic receptor P2X, ligand-gated ion channel 3), Nav1.7 (voltage-gated sodium channel alpha subunit 1.7), Nav1.8 (voltage-gated sodium channel alpha subunit 1.8), and Nav1.9 (voltage-gated sodium channel alpha subunit 1.9).

Nociceptors constitute a specialized subset of primary afferent neurons responsible for the detection, transduction, and propagation of noxious stimuli, which are defined as stimuli capable of inducing actual or potential tissue damage (Figure 1) [32]. As the primary sensory receptors for pain, nociceptors play a pivotal role in enabling the organism to detect and appropriately respond to harmful exogenous and endogenous signals [33]. The cell bodies of these neurons reside in peripheral sensory ganglia, mainly within the dorsal root ganglia (DRG) and trigeminal ganglia (TG) [34]. Each nociceptor projects a bifurcating axon, with one branch projecting peripherally to innervate cutaneous, musculoskeletal, and visceral structures, and the other projecting centrally to establish synaptic connections within defined laminar regions of the dorsal horn of the spinal cord [35].

Nociceptors exhibit considerable heterogeneity with respect to their conduction velocity, degree of myelination, and stimulus modality preference. Aδ fibers, characterized by thin myelination and intermediate conduction velocities (5–35 m/s), are typically associated with the rapid transmission of sharp, localized pain [36]. C fibers, which are unmyelinated and conduct more slowly (0.5–2 m/s), are involved in the mediation of dull, burning, and poorly localized pain sensations [37]. This physiological dichotomy corresponds to a molecular diversification of transducer proteins and ion channels, which confer polymodal or unimodal sensitivities to nociceptors [38]. Polymodal nociceptors are responsive to many noxious stimuli, while unimodal nociceptors are selectively sensitive to particular types of stimuli [39]. These functional phenotypes are regulated through developmental and activity-dependent processes and are influenced by the tissue environment [40]. Nociceptor transduction of injurious stimuli (mechanical, thermal, and chemical) is mediated by a complex array of ion channels and receptor proteins strategically localized at the peripheral terminal membranes, which convert many noxious modalities into electrical signals [8].

The physiological and molecular diversity of nociceptors underlies their varied functional roles in pain detection. This heterogeneity arises from the differential expression of specialized transducer proteins and ion channels, which facilitate the detection and transduction of a wide spectrum of noxious stimuli via distinct mechanisms [41]. TRP channels function as polymodal sensors essential for nociception [42]. TRPV1 responds to noxious heat (>42 °C), acidic pH, and capsaicin, allowing cation influx that depolarizes nociceptors and activates Ca^2+^-dependent pathways (PKC and CaMK), thereby sensitizing the channel and promoting hyperalgesia [43]. TRPA1 detects electrophilic irritants and inflammatory mediators, resulting in cation influx, nociceptor depolarization, and subsequent activation of PLC and MAPK signaling pathways, which enhance neurogenic inflammation and mechanical hypersensitivity [44]. TRPM8 is activated by cool temperatures and menthol, and modulates pain pathways, generally counteracting TRPV1-mediated heat pain [45]. Mechanical nociception is mediated mainly by Piezo1 and Piezo2 channels, which open in response to membrane stretch or shear forces, permitting rapid cation influx and membrane depolarization [46]. Piezo2 is abundantly expressed in DRG neurons and mediates the detection of light touch and mechanical nociception [47], whereas Piezo1 plays a contributory role under conditions of tissue stress [48]. Additional nociceptive inputs arise from acid-sensing ion channels (ASICs), which detect extracellular acidification by allowing Na^+^ and Ca^2+^ influx [49], and purinergic P2X receptors (notably P2X3), which become activated by extracellular ATP released from damaged cells, thereby facilitating the sustained transmission of chronic pain signals [50].

The combined ionic influx (principally Na^+^ and Ca^2+^) from these channels generates receptor potentials that activate VGSCs (Nav1.7, Nav1.8, and Nav1.9), amplifying depolarization into action potentials [51]. Nav1.7 controls action potential threshold [52], while Nav1.8 and Nav1.9 maintain action potential propagation in response to prolonged stimulation [53,54]. On the other hand, Ca^2+^ entry through VGCCs initiates several intracellular signaling cascades (such as cAMP, PKC, and MAPKs) that regulate ion channel function, trafficking, and gene expression [55].

**Figure 2:**
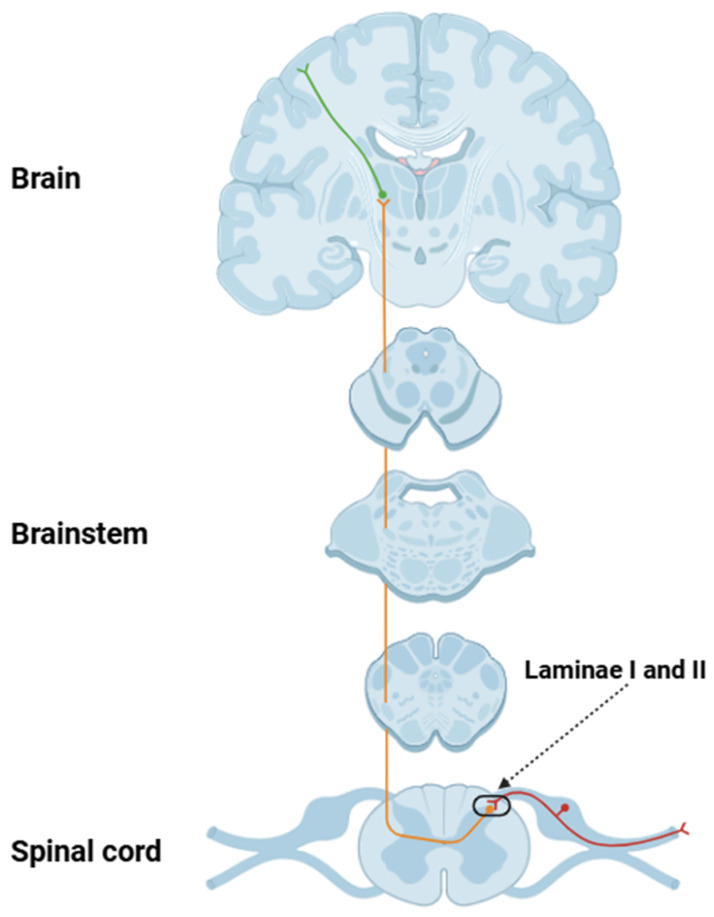
Schematic representation of the nociceptive transmission pathway. This figure illustrates the key anatomical components involved in the transmission of pain signals, beginning with the activation of peripheral nociceptors and culminating in the perception of pain in the cerebral cortex. It outlines the sequential steps of signal transduction, including primary afferent neuron activation, synaptic transmission in the dorsal horn of the spinal cord, ascending pathways (such as the spinothalamic tract), and the integration of nociceptive signals in the cerebral cortex. The red-colored neuron represents the first-order neuron, the orange one corresponds to the second-order neuron, and the green neuron denotes the third-order neuron.

Action potentials conducted along dorsal root axons primarily terminate in laminae I and II of the dorsal horn, forming synapses with second-order projection neurons and multiple interneuron types (Figure 2) [56]. Neurotransmission at these sites involves the release of excitatory neurotransmitters and neuromodulators (including glutamate, substance P, and CGRP) that modulate postsynaptic excitability and enhance nociceptive signal transmission to ascending spinal pathways such as the spinothalamic, spinoreticular, and spinoparabrachial tracts [57]. These ascending fibers project pain-related signals to a range of supraspinal structures (such as the thalamus, parabrachial nucleus, periaqueductal gray, amygdala, and somatosensory cortices), facilitating processing of the sensory-discriminative and affective-emotional components of pain [58].

A salient feature of nociceptors is their capacity for plasticity, a property that enables them to modulate their excitability and response thresholds in the context of inflammation, injury, or disease [59]. This neuroplastic adaptation constitutes the basis of peripheral sensitization, a process whereby exposure to inflammatory mediators such as PGE2, bradykinin, TNF-α, IL-1β, and NGF enhances the excitability of nociceptors, thereby promoting the emergence of hyperalgesia and allodynia in the context of injury or inflammation [60,61,62,63,64]. Sensitization involves post-translational modifications that enhance or modulate ion channel function (contributing to increased neuronal excitability) via phosphorylation by intracellular signaling cascades including PKA, PKC, and MAPKs [64] and longer-term transcriptional reprogramming that results in the upregulation of several pro-nociceptive genes [65]. In particular, NGF-TrkA signaling exerts potent effects on nociceptor gene expression, trafficking of ion channels to the membrane, and cytoskeletal dynamics, thereby contributing to both the acute sensitization and chronic hyperexcitability of these neurons [66].

Finally, under pathological conditions (particularly in neuropathic pain), nociceptors can undergo marked phenotypic transformations, leading to the generation of ectopic and spontaneous activity [67]. Additionally, interactions between injured nociceptors and immune elements, such as macrophages, T cells, and satellite glial cells, result in the establishment of a pro-inflammatory microenvironment that evokes nociceptive signaling [68]. This neuroimmune crosstalk is increasingly recognized as a key driver of pain chronification and involves modulation of cytokine release, chemokine signaling, and ion channel expression [69].

In summary, nociceptors comprise a highly specialized subset of peripheral sensory neurons, functionally specialized to detect, transduce, and transmit a wide array of potentially damaging stimuli [32]. Their molecular and functional plasticity not only facilitates adaptive responses within the pain pathway but also plays a central role in the development and maintenance of chronic pain states [59]. Advances in neurobiology, molecular genetics, and translational research are progressively clarifying the biology of nociceptors, thereby facilitating the design of highly effective analgesic strategies [70,71]. Defining nociceptor-specific signaling pathways and understanding their modulation in pathological contexts remain pivotal challenges in the effort to mitigate chronic pain and improve patient outcomes [72].

## 3. Structural and Molecular Changes of Nociceptors Following an Injury

Tissue injury induces a series of structural and molecular alterations in nociceptors. These alterations constitute an adaptive response intended to preserve tissue integrity and promote healing [73]; however, when dysregulated, they may facilitate the transition from acute to chronic pain [74]. These changes are not passive ramifications of injury, but constitute dynamic responses orchestrated by intrinsic cellular programs and extrinsic signals from the surrounding microenvironment [40].

### 3.1. Molecular-Level Transformations

In the peripheral axon, injured nociceptors initiate a regenerative response characterized by cytoskeletal remodeling, neurite outgrowth, and remodeling of target-specific axonal projections. From a molecular perspective, one of the earliest and most robust hallmarks of this process is the upregulation of a distinct set of regeneration-associated genes (RAGs) [75], like activating transcription factor 3 (ATF3) [76], small proline-rich protein 1A (Sprr1a) [77], growth-associated protein 43 (GAP-43) [78], and the transcription factor c-Jun [79]. These RAGs coordinate a transcriptional program that promotes axonal sprouting and structural reorganization essential for the extension of new growth cones and the establishment of novel axonal branches [75]. GAP-43 plays an essential role in modulating the dynamics of the cytoskeleton by interacting with actin filaments and regulating membrane plasticity at the growth cone, thereby facilitating axonal elongation [80]. In the same way, c-Jun acts as a master regulator of gene expression in injured neurons, coordinating pathways that govern cell survival, cytoskeletal remodeling, and growth cone formation [81,82,83]. Sprr1a, another key RAG, contributes to cytoskeletal stability and promotes neurite extension by interacting with intermediate filaments and actin networks [84].

There is a notable shift in the expression profile of integrins and cell adhesion molecules (CAMs), like neural cell adhesion molecule (NCAM) and L1 cell adhesion molecule (L1CAM) [85,86]. These adhesion molecules mediate some interactions between regenerating axons and the extracellular matrix (ECM) [87], as well as with surrounding Schwann cells and other non-neuronal elements in the peripheral nerve environment [88]. The altered expression and glycosylation patterns of NCAM and L1CAM strengthen axonal attachment and promote neurite outgrowth by activating intracellular signaling cascades involving focal adhesion kinase (FAK) and integrin-linked kinase (ILK) [89,90]. These kinases regulate cytoskeletal dynamics via downstream effectors such as the PI3K/Akt and MAPK/ERK pathways, which are essential for growth cone motility and directional guidance [89,90]. Moreover, the ECM itself undergoes remodeling after injury, with increased deposition of laminin, fibronectin, and tenascin-C, which serve as permissive substrates for axonal growth [91,92]. The interaction of integrins with ECM components not only provides mechanical support but also triggers intracellular signaling that strengthens regenerative capacity [93,94]. Schwann cells play a key role in this process by secreting neurotrophic factors, such as NGF and brain-derived neurotrophic factor (BDNF), which further stimulate RAG expression and cytoskeletal remodeling in nociceptors [95,96].

Recent advances in single-cell transcriptomics have substantially expanded our understanding of the cellular and molecular heterogeneity present within injured nociceptors. In particular, the application of single-cell RNA sequencing (scRNA-seq) has enabled the high-resolution characterization of transcriptional diversity across individual nociceptive neurons following injury [97]. These studies have shown the existence of distinct subpopulations of nociceptors, each characterized by unique expression profiles of RAGs, as well as varying capacities for axonal regeneration [98,99]. For instance, landmark investigations have demonstrated that injury-induced transcriptional responses are not uniform across all sensory neurons but instead are stratified according to subtype-specific molecular programs. This fact has allowed for the identification of discrete molecular signatures associated with axonal regeneration, inflammatory signaling pathways, and pain hypersensitivity [100,101].

Simultaneously, injury elicits pronounced changes in the expression and function of several ion channels in nociceptors, which critically contribute to the emergence of hyperexcitability and the maintenance of persistent pain states [102]. A significant feature of this plasticity is the upregulation and altered localization of VGSCs (Nav1.3, Nav1.7, Nav1.8, and Nav1.9). Nav1.3, typically absent in adult peripheral neurons, is re-expressed de novo following nerve injury and exhibits rapid repriming kinetics that facilitate high-frequency firing [103]. Nav1.7, a threshold channel crucial for amplifying subthreshold depolarizations, becomes overexpressed, thereby lowering the threshold for action potential initiation [104]. Nav1.8 and Nav1.9, mostly expressed in nociceptors, contribute to sustained depolarizing currents and are resistant to inactivation, promoting ectopic firing [105,106]. These sodium channelopathies collectively enhance excitability and underlie spontaneous discharges observed in neuropathic pain. Concomitant changes are shown in the expression of K^+^ channels (VGKCs), most notably a downregulation of delayed rectifier channels such as Kv1.2, which under normal conditions contribute to membrane repolarization and the termination of action potentials [107]. The reduction in outward K^+^ currents prolongs depolarization phases, thus facilitating aberrant firing patterns [108]. Ca^2+^ channel expression is also perturbed, particularly with an upregulation of VGCCs (like Cav2.2), which are integral to presynaptic Ca^2+^ influx and neurotransmitter vesicle exocytosis at central terminals in the dorsal horn [109]. The enhanced activity of Cav2.2 channels leads to increased release of excitatory neurotransmitters such as glutamate and substance P, amplifying nociceptive signaling in the CNS [110].

Optogenetic methodologies have become indispensable for dissecting the functional architecture of nociceptive circuits, owing to their exquisite spatial, temporal, and genetic precision [111,112]. In the optogenetic domain, targeted expression of excitatory opsins in genetically defined nociceptor subsets allows millisecond-scale control over action-potential firing. Illumination with the adequate wavelength can elicit or suppress pain-related behaviors in freely moving mice, establishing causal links between specific neuron populations and discrete components of the nociceptive experience [113]. Some studies utilized ChR2 in Nav1.7, Nav1.8, and Nav1.9-Cre-positive neurons to demonstrate that activation of peptidergic afferents is sufficient to trigger pain behaviors without concomitant tissue injury, thereby separating nociceptor activity from inflammatory confounds [114,115].

Additionally, TRP channels (especially TRPV1, TRPA1, and TRPM8) are critically involved in the modulation of sensory neuron responsiveness to thermal, chemical, and mechanical stimuli [116]. TRPV1, activated by thermal and chemical stimuli (such as capsaicin), undergoes sensitization via phosphorylation by kinases, including PKC and PKA, resulting in a lowered activation threshold [117]. TRPA1, activated by ROS and various environmental irritants, is likewise sensitized and frequently co-expressed with TRPV1, contributing to the development of mechanical and cold hyperalgesia [118]. TRPM8, a critical mediator of cold sensation, may also be upregulated under some pathological conditions, thereby contributing to the development of cold allodynia [45]. Chemogenetic strategies deploy engineered G-protein–coupled receptors (DREADDs) that respond only to otherwise inert ligands such as clozapine-N-oxide (CNO) or the more pharmacologically stable compound deschloroclozapine (DCZ) [119,120]. Some studies showed that chemogenetic silencing of TRPV1-lineage afferents attenuates inflammatory hyperalgesia, whereas their activation exacerbates mechanical allodynia, thereby highlighting the sufficiency and necessity of these neurons in driving persistent pain states [121].

These ion channel modifications are driven by a milieu of pro-inflammatory mediators released in the injured microenvironment. Pro-inflammatory cytokines such as TNF-α, IL-1β, and IL-6, along with neurotrophic factors (such as NGF) and lipid mediators like prostaglandins and bradykinin, bind to their respective receptors on nociceptor terminals [122,123,124,125,126]. These interactions initiate a cascade of intracellular signaling events, mainly involving MAPKs, PI3K/Akt, and PKC pathways [122,123,124,125,126]. All of these signaling cascades regulate transcriptional programs through activation of nuclear transcription factors like NF-κB and CREB, ultimately promoting the expression and post-translational modification of ion channels and receptors [127,128]. Moreover, these pathways influence axonal transport and membrane trafficking of several channel proteins, further enhancing nociceptor sensitivity and contributing to the persistent nature of chronic pain states [129].

Mitochondrial dynamics are also profoundly influenced by injury. Evidence suggests a shift toward increased mitochondrial fission, a process that facilitates the redistribution and transport of mitochondria along the axon [130]. This adaptation ensures the localized availability of ATP and other metabolic substrates at sites of axonal outgrowth and repair [131]. Enhanced mitochondrial transport from the soma to the distal axon terminals supports not only regenerative efforts but also sustains heightened synaptic activity, which is a hallmark of chronic pain states [132]. Moreover, injury-induced perturbations in the endoplasmic reticulum have been implicated in the pathophysiology of chronic nociception. Sustained endoplasmic reticulum stress induces the activation of the unfolded protein response (UPR), a fundamentally conserved cellular process responsible for the restoration [133]. Chronic activation of the UPR can result in maladaptive consequences, such as the modulation of ion channel expression, the induction of pro-inflammatory signaling cascades, and ultimately, the perpetuation of pain hypersensitivity [134,135,136].

**Figure 3:**
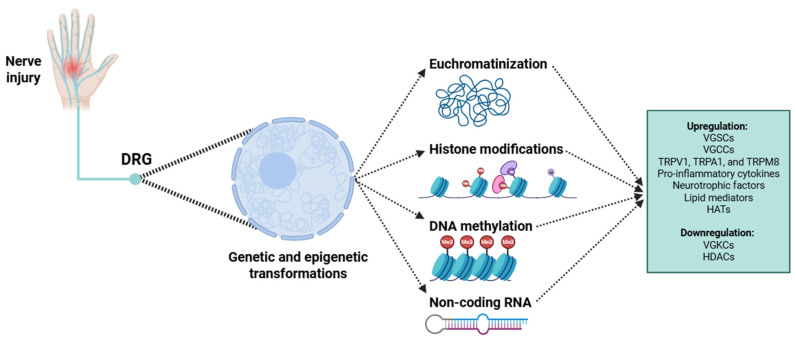
Graphical representation of genetic and epigenetic modifications in nociceptors induced by injury. Abbreviations: DRG (dorsal root ganglion), DNA (deoxyribonucleic acid), RNA (ribonucleic acid), VGSC (voltage-gated sodium channel), VGCC (voltage-gated calcium channel), TRPV1 (transient receptor potential vanilloid 1), TRPA1 (transient receptor potential ankyrin 1), TRPM8 (transient receptor potential melastatin 8), HAT (histone acetyltransferase), VGKC (voltage-gated potassium channel), and HDAC (histone deacetylase).

Finally, the long-term persistence of these structural and functional changes in nociceptors is reinforced by epigenetic modifications (Figure 3), which function as reversible mechanisms of gene regulation without altering the DNA sequence [137]. These modifications include histone acetylation, DNA methylation, and the modulatory influence of ncRNAs, all of which play key roles in driving the epigenetic reprogramming of nociceptor identity and function [137]. These epigenetic changes serve as a molecular interface through which several extracellular signals are transduced into stable changes in chromatin architecture and transcriptional output.

One of the earliest epigenetic responses involves histone acetylation. Activated transcription factors (such as CREB and NF-κB) recruit histone acetyltransferases (HATs) like p300/CBP to the promoters and enhancers of some pain-relevant genes, including *Scn9A*, *Scn10A*, *Trpv1*, *Bdnf*, and *Fos* [138]. Concomitantly, a reduction in the expression or activity of histone deacetylases (HDACs), including HDAC1, HDAC2, HDAC4, and HDAC5, has been observed in injured nociceptors [139]. In parallel with several histone modifications, DNA methylation plays a key role in the epigenetic reprogramming of injured nociceptors [140]. In rodent models of nerve injury, errant DNA methylation patterns have been observed, including hypermethylation of CpG islands in the promoter regions of genes encoding certain VGKCs (*Kcna2*, *Kcnq2*, and *Kcnd2*) [141]. This leads to reduced gene expression, impairing membrane repolarization capacity, and enhancing potential firing. Conversely, hypomethylation of pro-inflammatory or pro-nociceptive genes (such as *Trpa1*, *Il-6*, and *Atf3*) results in their overexpression [142]. This selective demethylation is often facilitated by TET family enzymes, mainly TET1 and TET3, which catalyze the oxidation of 5-methylcytosine to 5-hydroxymethylcytosine, initiating active DNA demethylation [143].

Another key layer of epigenetic regulation in injured nociceptors involves ncRNAs, miRNAs, lncRNAs, and circRNAs. For instance, miR-124 is downregulated in nociceptors after nerve injury, leading to disinhibition of key components of inflammatory pathways like TRAF6 and STAT3, and promoting the transcription of many pain-related genes [144]. In contrast, miR-21 is upregulated and contributes to nociceptor sensitization by targeting *Pten* and *Spry2*, negative regulators of MAPK signaling [145]. lncRNAs add a further dimension of complexity. The Kcna2 antisense RNA, transcribed from the opposite strand of the *Kcna2* gene, can interact directly with the corresponding mRNA or genomic DNA to repress transcription. This downregulation leads to a significant reduction in VGKCs activity, thereby enhancing neuronal excitability [146]. Moreover, lncRNAs like Neat1 and MALAT1 function as scaffolds for chromatin-modifying complexes. Neat1 facilitates the recruitment of EZH2 to specific gene promoters, where it mediates the trimethylation of H3K27me3, thereby contributing to transcriptional silencing [147]. These interactions silence anti-nociceptive or homeostatic genes, further cementing the chronic pain phenotype.

### 3.2. Structural-Level Transformations

The regenerative process involves extensive cytoskeletal remodeling, including the dynamic assembly and alignment of microtubules and actin filaments [148], which confer mechanical stability and spatial guidance for advancing growth cones [149]. Microtubule-associated proteins (MAPs), such as MAP1B and τ, undergo phosphorylation changes that reconfigure microtubule dynamics, facilitating axonal elongation and branching [150]. Actin cytoskeleton remodeling is regulated by several Rho GTPases (e.g., RhoA, Rac1, and Cdc42), which orchestrate growth cone dynamics and directional guidance by modulating cycles of actin polymerization and depolymerization [151].

**Figure 4:**
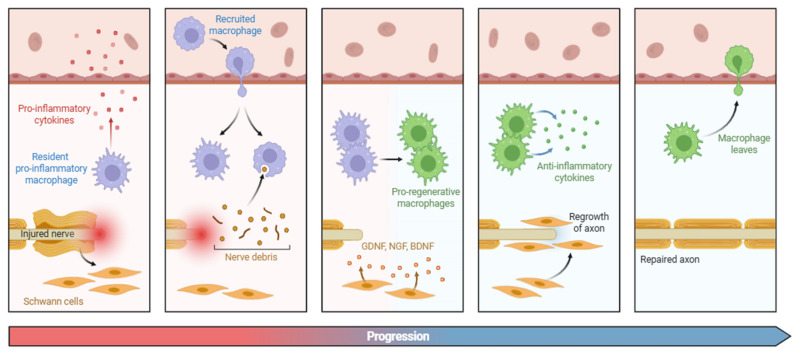
Schematic representation of the regenerative process of myelinated nociceptors. Following injury, resident macrophages release several pro-inflammatory cytokines (e.g., IL-1β, IL-6, and TNF-α), which recruit additional macrophages to the site of damage to promote axonal regeneration. In parallel, Schwann cells contribute to the regenerative environment by secreting neurotrophic factors such as GDNF, NGF, and BDNF, thereby supporting and accelerating the growth of injured axons. Abbreviations: GDNF (glial cell line-derived neurotrophic factor), NGF (nerve growth factor), and BDNF (brain-derived neurotrophic factor).

The surrounding non-neuronal cellular milieu exerts a profound influence on the initiation, maintenance, and exacerbation of neuroplastic and nociceptive processes following peripheral nerve injury (Figure 4) [152]. Schwann cells, in the peripheral nerve, and satellite glial cells (SGCs) in the DRG, undergo distinct phenotypic and functional reprogramming in response to injury, a process collectively known as reactive gliosis [153,154]. These adaptations are characterized by cellular proliferation and hypertrophy, increased expression of glial fibrillary acidic protein (GFAP), and augmented production and release of various pro-inflammatory and neuroactive mediators [155,156,157]. One of the distinguishing characteristics of this reactive phenotype is the increased expression of connexin 43 (Cx43), a gap junction protein that orchestrates direct cytoplasmic exchange between adjacent glial cells [158]. The upregulation of Cx43 reinforces intercellular coupling, thus synchronizing glial activity and amplifying neuroinflammatory signaling across the glial network [159]. This process not only supports the spread of Ca^2+^ waves and ATP-mediated signaling but also facilitates the paracrine and autocrine dissemination of some pro-inflammatory cytokines (such as IL-1β, IL-6, and TNF-α) [160]. Concurrently, Schwann cells and SGCs increase their release of neurotrophic factors, including BDNF, NGF, and glial cell line-derived neurotrophic factor (GDNF). Each of these factors exerts different effects on nociceptor excitability and plasticity [161].

Within the DRG, injury-induced alterations extend beyond peripheral axonal damage and encompass significant modifications in the somatic compartment of nociceptive neurons [162]. One of the earliest and most prominent cellular responses observed in the soma is a transformation in nuclear morphology. Following injury, the neuronal nucleus usually exhibits enhanced euchromatinization (Figure 3), evidenced by a relaxed and decondensed chromatin architecture [163,164]. This reflects an upregulation of transcriptional activity, as euchromatin is associated with an open chromatin state that enables active gene expression. Such transcriptional activation likely underlies the increased production of mRNAs required for both reparative and maladaptive cellular programs, including those associated with neuroinflammation and pain sensitization [165,166]. Simultaneously with chromatin remodeling, nucleolar hypertrophy is usually observed following peripheral nerve injury. The nucleolus, being the main site of rRNA transcription and ribosome assembly, shows morphological enlargement and upregulated function [167]. This morphological change reflects a substantial increase in ribosomal biogenesis, which is consistent with the heightened demand for protein synthesis necessary for axonal regeneration, cytoskeletal remodeling, and the synthesis of signaling molecules involved in nociceptive plasticity [168].

Centrally, nociceptive terminals located within the dorsal horn of the spinal cord undergo profound structural, molecular, and synaptic remodeling in response to peripheral nerve injury. One of the hallmark features of this reorganization is the aberrant sprouting of nociceptive C-fiber afferents into the dorsal horn laminae that are typically innervated by low-threshold mechanoreceptors [169]. Following injury, C-fiber terminals may extend into deeper laminae, such as lamina III, an area generally associated with innocuous tactile input conveyed by myelinated Aβ fibers [170]. This ectopic innervation enables nociceptors to form de novo synaptic contacts with interneurons and projection neurons that are not typically involved in pain processing under physiological conditions [171]. The resulting convergence of nociceptive and mechanoreceptive afferents onto common postsynaptic targets constitutes a critical mechanism underlying mechanical allodynia [172]. Injured nociceptors contribute significantly to this hyperexcitable state by releasing a suite of pro-nociceptive neurotransmitters and neuromodulators, such as glutamate, substance P, and CGRP [173]. These molecules promote central sensitization, a pathophysiological process characterized by increased excitability and synaptic efficacy of dorsal horn neurons [174].

Taken together, the structural transformations of nociceptors following injury are extensive and involve remodeling at the molecular and cellular levels. These changes extend from the peripheral terminals through the axon and soma to the central terminals in the spinal cord, forming a continuum of plasticity that supports the transition from acute to chronic pain. Understanding the precise mechanisms that govern these structural alterations holds promise for the development of targeted therapeutic strategies aimed at reversing or preventing pathological pain states.

## 4. Conclusions

In summary, peripheral nerve injury triggers many structural and molecular modifications in nociceptors that profoundly alter their functional properties and contribute to the pathogenesis of chronic pain. At the structural level, injury induces axonal sprouting, synaptic reorganization, and phenotypic shifts in nociceptors, including altered soma size and changes in connectivity with spinal interneurons. These changes are accompanied by a dynamic molecular landscape characterized by transcriptional reprogramming, epigenetic remodeling, and post-transcriptional regulation.

The molecular and cellular changes initiated by peripheral nerve damage extend beyond the acute phase of nociceptor sensitization, playing integral roles in the maintenance and amplification of chronic pain states. These injury-induced signatures, which encompass diverse classes of transcripts, proteins, post-translational modifications, and epigenetic marks, represent a rich reservoir of candidate biomarkers and actionable therapeutic targets with substantial translational potential [31]. Rather than viewing these molecular profiles solely as correlates of disease, emerging evidence suggests they might function as mechanistic drivers of pain chronification and as predictive indicators of therapeutic responsiveness.

Transcriptional reprogramming in injured nociceptors can be quantitatively profiled using many RNA sequencing approaches [100,101]. Such gene expression signatures have already begun to distinguish neuropathic pain subtypes linked to specific etiologies, such as diabetic neuropathy, traumatic nerve injury, or chemotherapy-induced peripheral neuropathy [175]. Epigenetic landscapes offer an additional molecular framework that could serve as reliable biomarkers of chronic pain states [176].

In addition to their diagnostic utility, these molecular profiles offer therapeutic leverage points. One promising avenue involves the modulation of ion channel expression and function. Given the key role of VGSCs, VGCCs, and TRP channels in regulating nociceptor excitability, pharmacological agents or gene-silencing approaches (such as siRNAs, antisense oligonucleotides, or CRISPR-Cas-based repressors) targeting these channels are under intense investigation [177]. Crucially, the specificity of these therapeutic interventions might be enhanced through integration with many molecular diagnostic approaches that confirm the upregulation of the corresponding targets in a given patient.

Similarly, the disruption of several pro-inflammatory signaling pathways constitutes an additional therapeutic strategy grounded in molecular pathology. Small molecule inhibitors of cytokine receptors (such as IL-1βR and TNFR1), chemokine antagonists (such as CCR2 and CXCR3), or intracellular kinases (e.g., MAPK, JNK, and IKKβ) have shown efficacy in preclinical models and are advancing through various stages of clinical development [178,179,180]. These interventions are designed to disrupt the pathological neuroimmune crosstalk that sustains nociceptor hyperexcitability and glial activation within the injured nervous system.

Epigenetic therapeutics constitute a key innovative frontier. HDAC inhibitors, DNA methyltransferase inhibitors, and RNA-based epigenetic modulators have demonstrated the capacity to reverse injury-induced transcriptional changes in nociceptors and restore homeostatic gene expression patterns. Such pharmacological agents, though still largely experimental in the context of pain, might offer the potential to “reset” nociceptor phenotype toward a non-sensitized state [181,182]. On the other hand, the development of targeted delivery systems (e.g., nanoparticle-based vectors or viral gene delivery platforms) might enable tissue- or cell-specific epigenetic interventions, thereby minimizing systemic side effects [183,184,185].

Importantly, these molecular strategies must be integrated into a framework of precision medicine. Chronic pain is a heterogeneous condition with many underlying mechanisms, and the identification of molecular endophenotypes within patient populations is essential for matching individuals with the most appropriate therapeutic modality [186]. Molecular biomarkers not only enable stratification of patients into mechanistically distinct subgroups but may also provide dynamic indicators of treatment efficacy, allowing real-time monitoring and adjustment of therapeutic regimens [187]. In this context, the convergence of molecular biology, systems neuroscience, and clinical pain research generates unprecedented opportunities. Multi-omics integration (combining transcriptomic, epigenomic, proteomic, and metabolomic data) can yield holistic disease signatures that more accurately reflect the complexity of chronic pain states. Coupled with machine learning and bioinformatics tools, these approaches may uncover latent molecular patterns that escape detection through conventional analyses.

Together, these findings illustrate that the nociceptor is not a passive conduit for pain signals but an active participant in the development of chronic pain through injury-induced neuroplasticity. Future research should focus on identifying the temporal sequence of these changes, their cell-type specificity, and their reversibility, which will be essential for the design of precision medicine approaches tailored to individual pain phenotypes.

Finally, this review offers a novel contribution by systematically delineating injury-induced structural and molecular adaptations that are specific to nociceptors. In contrast to previous studies that have focused on broader neuronal or systemic responses to injury, this article emphasizes the cellular remodeling and transcriptional reprogramming occurring within nociceptors. By providing a focused synthesis of these neuron-specific modifications, this review refines current understanding of pain mechanisms.

## Data Availability

Not applicable. No new data were generated.

## Abbreviations

The following abbreviations are used in this manuscript:

ASIC	Acid-sensing ion channel
ATF3	Activating transcription factor 3
ATP	Adenosine triphosphate
BDNF	Brain-derived neurotrophic factor
CAM	Cell adhesion molecule
CaMK	Calcium/calmodulin-dependent protein kinase
cAMP	Cyclic adenosine monophosphate
Cav2.2	Voltage-gated calcium channel, subtype 2.2
CCL2	Chemokine (C-C motif) ligand 2
CCR2	C-C Motif chemokine receptor 2
Cdc42	Cell division cycle 42
CGRP	Calcitonin gene-related peptide
ChR2	Channelrhodopsin-2
circRNA	Circular RNA
c-Jun	Cellular Jun (a component of the AP-1 transcription factor complex)
CNO	Clozapine-N-oxide
CNS	Central nervous system
CREB	cAMP response element-binding protein
CRISPR	Clustered regularly interspaced short palindromic repeats
CXCR2	C-X-C Motif chemokine receptor 2
Cx43	Connexin 43
DCZ	Deschloroclozapine
DNA	Deoxyribonucleic acid
DREADD	Designer receptor exclusively activated by designer drugs
DRG	Dorsal root ganglion
ECM	Extracellular matrix
ERK	Extracellular signal-regulated kinase
EZH2	Enhancer of zeste homolog 2
FAK	Focal adhesion kinase
GAP-43	Growth-associated protein 43
GCN5	General control non-derepressible 5
GDNF	Glial cell line-derived neurotrophic factor
GFAP	Glial Fibrillary Acidic Protein
H3K27me3	Trimethylation of lysine 27 on histone H3
HAT	Histone acetyltransferase
HDAC	Histone deacetylase
HDAC1	Histone deacetylase 1
HDAC2	Histone deacetylase 2
HDAC4	Histone deacetylase 4
HDAC5	Histone deacetylase 5
IASP	International Association for the Study of Pain
IKKβ	IκB kinase beta
IL-1β	Interleukin 1 beta
IL-1βR	Interleukin 1 beta receptor
IL-6	Interleukin 6
ILK	Integrin-linked kinase
JNK	c-Jun N-terminal kinase
Kcna2	Potassium voltage-gated channel subfamily A member 2
Kcnd2	Potassium voltage-gated channel subfamily D member 2
Kcnq2	Potassium voltage-gated channel subfamily Q member 2
L1CAM	L1 cell adhesion molecule
lncRNA	Long non-coding RNA
MALAT1	Metastasis-associated lung adenocarcinoma transcript 1
MAP	Microtubule-associated protein
MAP1B	Microtubule-associated protein 1B
MAPK	Mitogen-activated protein kinase
miRNA	MicroRNA
mRNA	Messenger ribonucleic acid
Nav1.7	Voltage-gated sodium channel, subtype 1.7
Nav1.8	Voltage-gated sodium channel, subtype 1.8
Nav1.9	Voltage-gated sodium channel, subtype 1.9
NCAM	Neural cell adhesion molecule
ncRNA	Non-coding RNA
Neat1	Nuclear paraspeckle assembly transcript 1
NF-κB	Nuclear factor kappa-light-chain-enhancer of activated B cells
NGF	Nerve growth factor
P2X	Purinergic receptor P2X
P2X3	Purinergic Receptor P2X, ligand-gated ion channel 3
p300/CBP	E1A binding protein p300/CREB-binding protein
PGE2	Prostaglandin E2
PI3K/Akt	Phosphoinositide 3-kinase/protein kinase B
Piezo	Piezo-type mechanosensitive ion channel
Piezo1	Piezo-type mechanosensitive ion channel 1
Piezo2	Piezo-type mechanosensitive ion channel 2
PKA	Protein kinase A
PKC	Protein kinase C
PLC	Phospholipase C
Pten	Phosphatase and tensin homolog
Rac1	Ras-related C3 botulinum toxin substrate 1
RAG	Regeneration-associated gene
Rho GTPase	Ras homolog family of GTPases
RhoA	Ras homolog gene family member A
ROS	Reactive oxygen species
rRNA	Ribosomal ribonucleic acid
scRNA-seq	Single-cell RNA-sequencing
SGC	Satellite glial cell
Sprr1a	Small proline-rich protein 1A
Spry2	Sprouty RTK signaling antagonist 2
STAT3	Signal transducer and activator of transcription 3
TET	Ten-eleven translocation (dioxygenase family)
TET1	Ten-eleven translocation methylcytosine dioxygenase 1
TET3	Ten-eleven translocation methylcytosine dioxygenase 3
TG	Trigeminal ganglion
TNF-α	Tumor necrosis factor alpha
TNFR1	Tumor necrosis factor receptor 1
TRAF6	TNF receptor-associated factor 6
TrkA	Tropomyosin receptor kinase A
TRP	Transient receptor potential
TRPA1	Transient receptor potential ankyrin 1
TRPM8	Transient receptor potential melastatin 8
TRPV1	Transient receptor potential vanilloid 1
UPR	Unfolded protein response
VGCC	Voltage-gated calcium channel
VGKC	Voltage-gated potassium channel
VGSC	Voltage-gated sodium channel
